# Spherical model for Minimalist Machine Learning paradigm in handling complex databases

**DOI:** 10.3389/frai.2025.1521063

**Published:** 2025-02-14

**Authors:** Raúl Jimenez-Cruz, Cornelio Yáñez-Márquez, Miguel Gonzalez-Mendoza, Yenni Villuendas-Rey, Raúl Monroy

**Affiliations:** ^1^Tecnologico de Monterrey, School of Engineering and Sciences, Monterrey, Mexico; ^2^Smart Computing Laboratory, Centro de Investigación en Computación, Instituto Politecnico Nacional, Mexico, Mexico

**Keywords:** classification, machine learning, pattern recognition, Minimalist Machine Learning, pattern classification

## Abstract

This paper presents the development of the N-Spherical Minimalist Machine Learning (MML) classifier, an innovative model within the Minimalist Machine Learning paradigm. Using N-spherical coordinates and concepts from metaheuristics and associative models, this classifier effectively addresses challenges such as data dimensionality and class imbalance in complex datasets. Performance evaluations using the F1 measure and balanced accuracy demonstrate its superior efficiency and robustness compared to state-of-the-art classifiers. Statistical validation is conducted using the Friedman and Holm tests. Although currently limited to binary classification, this work highlights the potential of minimalist approaches in machine learning for classification of highly dimensional and imbalanced data. Future extensions aim to include multi-class problems and mechanisms for handling categorical data.

## 1 Introduction

Research into the best intelligent classifier has long been recognized as a formidable challenge, especially after the introduction of “The No Free Lunch Theorem” in 1997 (Wolpert and Macready, 1997; Wolpert, 2002). This theorem underscores the impossibility of designing a universal classifier that excels across all types of datasets. In response to these challenges, this study introduces the N-Spherical Minimalist Machine Learning (MML) classifier, which simplifies the representation of high-dimensional data using spherical coordinates and enhances classification performance by addressing class imbalance and data complexity. This innovative model combines metaheuristics with N-spherical coordinate transformations to provide an efficient solution within the Minimalist Machine Learning (MML) paradigm.

The main problem addressed in this research involves overcoming data complexity issues, including high dimensionality and class imbalance, which pose significant obstacles in real-world datasets. Our approach focuses on developing the N-Spherical MML classifier, a novel model grounded in the MML paradigm. This paradigm operates under the hypothesis that re-distributing the spatial positioning of patterns through coordinate transformation can enhance classification outcomes.

Moreover, this approach integrates a unique feature selection mechanism to determine both the optimal number of features and their relative importance, thereby reducing dataset complexity. Our pioneering methodology, which fuses the MML framework with non-Cartesian coordinates and an innovative attribute selection strategy, represents a significant advancement in the field. The combination of these elements underscores the potential of minimalist approaches in addressing complex challenges in machine learning, paving the way for future research in multi-class classification and the management of diverse data types.

## 2 Materials and methods

This section examines existing methods in machine learning, focusing on their application to pattern classification and reduction of imbalance. By analyzing recent advances and their limitations, we establish the foundation for introducing the novel contributions of the N-Spherical MML model.

### 2.1 Metaheuristics

Nature serves as a vast optimization mechanism, where countless species evolve, demonstrating refined survival abilities over time. Inspired by nature's behavior, evolutionary computing has experienced remarkable growth. This field mimics natural processes through highly adaptive algorithms that solve a wide range of problems using metaheuristics. These algorithms randomly generate numerous potential solutions, evolving through local search operators, information exchange between individuals, and random mutations.

Researchers in metaheuristics have drawn inspiration from various natural and social phenomena, as well as the behaviors of living species, including genetics, immune systems, ant colonies, bees, bats, swarms, music, fireflies, chemical reactions, fish, and birds. For instance, in García-calvo et al. (2018), a variant of genetic algorithms, one of the oldest and most renowned metaheuristics, employs granulated structures for effective feature selection. Yelghi and Köse (2018) and Zhang et al. (2018) explore metaheuristic variants inspired by fireflies and gray wolves for optimization. Storn and Price (1997) describes a straightforward yet effective metaheuristic called Differential Evolution, which employs randomly diverse individuals to generate offspring through simple combinations within the original population.

In our work, we chose to leverage Differential Evolution to enhance our proposed model by autonomously adjusting two parameters involved in both the learning and classification phases. This decision was made due to the rapid convergence, robustness, and simplicity of this metaheuristic.

### 2.2 Class imbalance

Class imbalance in databases poses a significant challenge, affecting the performance of pattern classifiers. Alongside class overlap, outliers, and mixed or missing data, class imbalance presents adverse scenarios that require effective strategies when developing new classifier models.

In Tang and He (2017), two sampling approaches for learning in imbalanced databases are presented: under-sampling and over-sampling. The study also introduces a novel metric called the Generalized Imbalance Ratio (GIR), achieving success by bagging multiple known classifiers.

Similarly, Hu et al. (2018) addresses class imbalance by maximizing the area under the curve (AUC) and proposing a new learning algorithm for imbalanced data called Kernelized Online Imbalanced Learning (KOIL). Another approach is presented in Liu et al. (2018), where a solution to the multiple criteria problem is introduced through example assignment, while Maldonado and López (2018) addresses high dimensionality challenges and uses Support Vector Machines (SVM) for feature selection.

Additionally, Wong et al. (2018) addresses the class imbalance problem using a hybrid method that combines metaheuristics with evaluations in three of the most prominent classifiers in the field: SVM, C4.5, and k-NN. Lastly, Das et al. (2018) provides an extensive survey on data complexity issues (termed “irregularities” by the authors), offering numerous research avenues for future exploration. These ideas have inspired certain aspects of the development of our proposed model.

In summary, while existing methods have laid the groundwork for advancements in machine learning, they often fall short in addressing the specific challenges posed by high-dimensional, imbalanced datasets. The N-Spherical MML model, with its innovative integration of metaheuristics, class imbalance handling, and dimensionality reduction, represents a significant step forward in this domain.

### 2.3 Minimalist Machine Learning paradigm

Minimalist Machine Learning (MML) is an innovative paradigm designed for intelligent pattern classification tasks. This paradigm is grounded in the notion that the input receives a dataset D consisting of N patterns with n attributes distributed across m classes.

Example: Suppose we have a dataset with two classes and various attributes. The MML approach simplifies this by focusing on essential attributes and transforming the data into a spherical coordinate system, which aids in the classification process.


(1) $$\begin{array}{l} \text{If }k\in N,k<=N\to x^{k}=\left[x_{1}^{k},x_{2}^{k},...,x_{n}^{k}\right] \end{array}$$

Definitions:

- k represents the k-th pattern in the dataset D.
- n denotes the n-th attribute in the dataset D.

The goal is to produce a graphical representation on the Cartesian plane that depicts the classes and includes a straight line minimizing errors or ideally separating the two classes.

Once the dataset D is inputted, a validation method is applied to obtain two disjoint sets. The training phase involves finding a subset of features to which a set of operations can be applied in both the training and test sets, but separately in each set.

The classification phase of MML entails taking a pattern from the test set and checking whether it lies above or below the horizontal line. Ideally, the output is expected to be a graph on the Cartesian plane where the two classes are represented, along with a horizontal line that minimizes errors separating these two classes.

Building on the aforementioned, it can be stated that the MML paradigm has a central premise that there exists a family of subsets of the n attributes for which there is a set of simple operations applied in a specific order that yields the desired representation on the Cartesian plane.

Therefore, it can be said that the family of subsets, the operations to be performed (applied to every pattern in the training and test sets), and the order of application define a unique algorithm within the MML paradigm.

### 2.4 Spherical coordinates in N dimensions

Polar coordinates are a coordinate system determined by a distance and an angle (Anton et al., 2010). Like the Cartesian coordinate system, this system is determined by a point O, also called the origin. Following the logic of Cartesian coordinates, the ordered pair for polar coordinates is denoted as follows (r, θ), where r is the distance between the origin O and the point P, and θ is the angle formed between the polar axis and a line from O to P.

Based on this concept, polar coordinates can be expanded to N dimensions, which change names as the dimension grows. When implemented in 3 dimensions, they are called spherical coordinates, and when expanded beyond 3 dimensions, they are termed hyperspherical coordinates. An N-dimensional sphere is a generalization of a sphere in a hyperplane.

In 1960, lecture notes were published (Blumenson, 1960) that implemented a method of generalization for three-dimensional spherical coordinates without requiring geometric intuition. Basically, according to the definition in the publication, transitioning from Cartesian coordinates in n dimensions to spherical coordinates in n dimensions works as follows:

Given a point x in Cartesian space with n components (x1, x2, x3, …, xn), it is possible to transform the components of the point x into components in hyperspherical space.


(2) $$\begin{array}{l} \left(x_{1},x_{2},x_{3},\ldots ,x_{n}\right)\to \left(r,\alpha_{1},\alpha_{2},\ldots ,\alpha_{n-1}\right) \end{array}$$

Where:

- (*x*_1_, *x*_2_, *x*_3_, …, *x*_*n*_) : They are each of the n components of x in Cartesian space.
- r: It is the radius from the origin of the sphere to the point in n-spherical space.
- (α_1_, α_2_, …, α_*n*−1_) : They are the n-1 dimensional angles of the hypersphere.

To obtain each component in N-spherical space, the following equations developed in [33] are used:


(3) $$\begin{array}{l} r=\sqrt{{\left(x_{1}\right)}^{2}+{\left(x_{2}\right)}^{2}+...+{\left(x_{n-1}\right)}^{2}+{\left(x_{n}\right)}^{2}} \end{array}$$

(4) $$\begin{array}{l} \alpha_{1}=arcos\left(\frac{x_{1}}{\sqrt{{\left(x_{1}\right)}^{2}+{\left(x_{2}\right)}^{2}+...+{\left(x_{n-1}\right)}^{2}+{\left(x_{n}\right)}^{2}}}\right) \end{array}$$

(5) $$\begin{array}{l} \alpha_{2}=arcos\left(\frac{x_{2}}{\sqrt{{\left(x_{2}\right)}^{2}+{\left(x_{3}\right)}^{2}+...+{\left(x_{n-1}\right)}^{2}+{\left(x_{n}\right)}^{2}}}\right) \end{array}$$

(6) $$\begin{array}{l} \alpha_{n-2}=arcos\left(\frac{x_{n-2}}{\sqrt{{\left(x_{n-2}\right)}^{2}+{\left(x_{n-1}\right)}^{2}+{\left(x_{n}\right)}^{2}}}\right) \end{array}$$

(7) $$\begin{array}{l} \alpha_{n-1}=arcos(\frac{x_{n-1}}{\sqrt{({\left(x_{n-1}\right)}^{2}+{\left(x_{n}\right)}^{2}}}) \end{array}$$

As shown, the primary component of N-spherical coordinates is the radius r (Equation 3), which determines the distance of a point x from the origin in the N-dimensional space. This component encapsulates the magnitude of the point's position relative to the center of the coordinate system.

The remaining components (Equations 4–7) are angular coordinates that define the specific orientation of the point within the spherical system. These angles provide additional geometric information by describing the point's position along different axes of the N-dimensional space. Together, the radius and angles form a comprehensive representation of the point, allowing for a more nuanced understanding of its spatial relationship to other points in the dataset. This coordinate system plays a pivotal role in the N-Spherical MML model, as it enables the transformation of Cartesian data into a form that better highlights separability between classes.

### 2.5 Datasets

This section describes the datasets used in our proposed model and at the final of this section we have a Table 1 where is resumed all important data of the datasets:

- Leukemia dataset (Golub et al., 1999): Microarray dataset containing gene expression data related to leukemia. It consists of 38 patterns, with 27 patterns in the “Acute Lymphoblastic Leukemia” (ALL) class and 11 in the “Acute Myelogenous Leukemia” (AML) class. After feature selection, the dataset contains 3,052 genes.
- Nutt dataset (Nutt et al., 2003): Dataset comprising 28 patterns of brain cancer patients, divided into “Classic Glioblastomas” (CG) and “Non-Classic Glioblastomas” (NCG) classes, each with 14 patterns. This dataset contains 1071 genes.
- Lymphoma dataset (OpenML, Unknown)^1^: Contains 45 patterns classified into “Germinal Center B-cell Lymphoma” (GCL) and “Anaplastic Cell Lymphoma” (ACL) classes, with 22 and 23 patterns, respectively.
- Lung dataset (Sun, 2014): Consists of 85 patterns related to lung cancer, with 62 patterns in the “Small cell lung cancer” (SCLC) class and 24 in the “Non-small cell lung cancer” (NSCLC) class.
- AP Endometrium Prostate dataset (Stiglic and Kokol, 2010e): Gene expression dataset linking endometrial and prostate cancer. It contains 130 patterns, with 61 in the “Endometrium” class and 69 in the “Prostate” class.
- Covid-19 Kaggle dataset (Harikrishnan, 2020): Used for classifying COVID-19 cases. This dataset comprises 5,434 patterns, with 4,383 patterns labeled as “Yes” and 1,051 patterns as “No.”
- AP Ovary Lung dataset (Stiglic and Kokol, 2010e): Contains gene expression data associating ovarian and lung cancer. It consists of 324 patterns, with 198 in the “Ovary” class and 126 in the “Lung” class.
- AP Breast Uterus dataset (Stiglic and Kokol, 2010b): Gene expression dataset relating breast and uterine cancer. It includes 468 patterns, with 344 in the “Breast” class and 124 in the “Uterus” class.
- AP Breast Ovary dataset (Stiglic and Kokol, 2010a): Gene expression dataset linking breast and ovarian cancer. It comprises 542 patterns, with 344 in the “Ovary” class and 198 in the “Breast” class.
- AP Lung Kidney dataset (Stiglic and Kokol, 2010d): Contains gene expression data associating lung and kidney cancer. It consists of 386 patterns, with 260 in the “Kidney” class and 126 in the “Lung” class.
- AP Endometrium Kidney dataset (Stiglic and Kokol, 2010c): Gene expression dataset linking endometrial and kidney cancer. It comprises 321 patterns, with 61 in the “Endometrium” class and 260 in the “Kidney” class.
- Diabetic Mellitus dataset (Abdulrahman, 2019): Used to classify diabetes mellitus cases. This dataset contains 281 patterns, with 99 in the “positive” class and 182 in the “negative” class.
- Brain Cancer Kaggle dataset (JillaniSoftTech, Unknown)^2^: Used for classifying brain cancer tumors. It consists of 36 patterns, with 18 in the “Normal” class and 18 in the “Tumor” class.

**Table 1:** Datasets description.

**Dataset name**	**Number of patterns**	**Number of attributes**	**Patterns at positive class**	**Patterns at negative class**	**Imbalance ratio**
Leukemia	38	3,052	27	11	2.45
Nutt	28	1,071	14	14	1
Lymphoma	45	4,026	22	23	1.04
Lung	85	7,130	62	24	2.58
AP Endometrium Prostate	130	10,936	61	69	1.13
Covid-19 Kaggle	5,434	20	4,383	1,051	4.17
AP Ovary Lung	324	10,936	198	126	1.57
AP Breast Uterus	468	10,936	344	124	2.77
AP Breast Ovary	542	10,936	344	198	1.73
AP Lung Kidney	386	10,936	260	126	2.06
AP Endometrium Kidney	321	10,936	61	260	4.26
Diabetic Mellitus	281	97	99	182	1.83
Brain Cancer Kaggle	36	7,465	18	18	1

The resumed data about every dataset is shown in Table 1.

### 2.6 State-of-the-art classifiers for comparison

WEKA (Das et al., 2018) is a data analysis and machine learning platform written in Java. Developed by the University of Waikato in New Zealand, this platform offers a wide range of algorithms for data analysis and predictive modeling, including regression, classification, and clustering tasks.

The software allows datasets to be preprocessed, if necessary, and inserted into a learning scheme to analyze the results and performance of a selected classifier. The main objective of using WEKA in this study is to compare the results obtained between the proposed model and a diverse collection of well-known classifiers. In the following, we describe the algorithms used in this platform:

- Naïve Bayes Algorithm: Naïve Bayes (Kurzyński, 1988) is a classification algorithm based on the application of Bayes' theorem, which calculates the probability of a hypothesis given the evidence. It assumes that the features are conditionally independent, given the class label. Instance-Based Classifier (IBK).
- The Instance-Based Classifier (IBK): Aha et al. (1991) is an enhanced version of the K-Nearest Neighbors (KNN) algorithm. Although KNN can only handle numerical values, IBK can work with both categorical and numerical values, as well as handling missing values using an implemented measure called the heterogeneous Euclidean-overlap measure (HEOM).
- Logistic regression: Logistic regression is a classification algorithm used for predictive modeling when the dependent variable (target) is categorical. It is particularly useful for binary classification problems, where the outcome variable has only two possible classes or states (e.g., 0 or 1, yes or no).
- Sequential Minimal Optimization (SMO): SMO is a popular algorithm used for training Support Vector Machines (SVM), a powerful model for pattern classification (Cortes and Vapnik, 1995). SVMs are based on a well-founded theory and aim to find the hyperplane that maximizes the margin of separation between classes or nearly separates them with a slight margin of error. The key idea behind SVMs is to transform the input data into a higher-dimensional space where it can be linearly separated. This transformation is achieved using a kernel function selected by the designer, which helps in finding the support vectors that define the created hyperplanes.
- Decision trees: Decision trees (Rodner and Denzler, 2011) are well-known supervised learning algorithms. They have a tree-like structure where each internal node or leaf represents a feature, and the final leaf nodes constitute the classification prediction. All nodes are connected by branches that represent simple if-then-else rules inferred from the data features.
- Multi-Layer Perceptron: The multi-layer perceptron (Rumelhart et al., 1986; Hall et al., 2009) is an artificial neural network that can be used as a classifier, representing an enhanced version of the simple perceptron. The key feature of this algorithm is its multiple layers, which enable it to tackle nonlinear problems. While neural networks offer numerous advantages, such as high performance and suitability for tasks like image classification, they also come with limitations. One significant drawback is that if the model is not properly trained, it tends to produce highly inaccurate results. Additionally, the optimization functions used in training neural networks often seek only local minima, which can lead to premature termination of the training process, even without reaching the desired error threshold set by the designer.

Another significant challenge is the considerable training and classification time required, particularly when working with high-dimensional datasets like those in this study. These computational demands can become a bottleneck, especially in scenarios where rapid model deployment or frequent retraining is necessary. To address this, our implementation in WEKA was optimized by configuring the Multilayer- perceptron algorithm with five hidden layers. This adjustment was made after observing that the default configuration resulted in excessively long training times, making the process impractical for large-scale experiments.

By reducing the number of hidden layers, the algorithm achieves a balance between computational efficiency and model performance, ensuring that it remains suitable for real-world applications. Despite the inherent challenges posed by high-dimensional data, this optimization retains the model's ability to deliver reliable and accurate classification results. Furthermore, this adjustment highlights the importance of fine-tuning hyperparameters to meet specific performance and scalability requirements, particularly in machine learning tasks involving complex datasets.

## 3 Results

To measure the performance of the proposed N-Spherical MML model, preprocessing steps were implemented to ensure consistency across datasets. For datasets with missing values, imputation techniques were applied, while label coding was used to handle categorical values. The programming and implementation were carried out using MATLAB, executed on a MacBook Pro laptop equipped with an M1 Pro processor and 16 GB of RAM. Notably, no special MATLAB functions or toolboxes were used; all implementations were developed from scratch using custom code.

The selected performance measure was balanced accuracy, which gives equal importance to both classes. Once this performance measure was established, statistical tests were conducted to comprehensively evaluate the results. Specifically, **the Friedman test**, a nonparametric statistical test, was applied to verify whether statistically significant differences exist between the performances of the classifiers. To identify where these differences occur, **the Holm Test (*Post-Hoc*)** was used. A significance level of α = 0.05 was considered, ensuring confidence in 95%. Rejection of the null hypothesis indicates significant differences among the classification algorithms.

### 3.1 N-Spherical MML model

This section presents the most relevant part of this work: the methodology for constructing the proposed model. It begins with the conceptual foundations and progresses to the complete assembly of the model with the purpose of understanding the complete operation.

The proposed model is built on a simple yet solid conceptual basis, combining the methods of Minimalist Machine Learning (**MML**) with the application of N-spherical coordinates. Additionally, a significant contribution to the proposed algorithm was implemented, in which a selection of the most relevant attributes is made using a method that we call “Twice mean” or T-means.

The hypothesis posits that points plotted in Cartesian space with a certain distribution will exhibit a distinct distribution in spherical space. With this premise in mind, the following question arises.

If MML involves separating two classes in the Cartesian plane using a horizontal line, what will happen in a spherical space?

The answer is straightforward: instead of separating the classes with a horizontal line in the polar plane, the classes can be separated by a circle of radius rm, which we will call a “circumference” in the graphs and a “radius” when referring to the calculated value of these circumferences. One class lies within this circumference, and the other class lies outside this circumference of radius rm.

This classifier assumes that the dataset D is initially represented in Cartesian coordinates, with each feature of the patterns corresponding to a component in Cartesian space. For instance, in the “Iris Dataset” (Dua and Taniskidou, 2017), each pattern has four features, representing a point in a 4-dimensional Cartesian space. This assumption enables us to convert each pattern from Cartesian coordinates to N-dimensional spherical coordinates, resulting in a newly derived dataset that can offer different insights and improved model performance.

The proposed model is based on this premise, where the search for the most relevant attributes for each dataset will be performed to train the model in the training phase and test the model in the classification phase. Additionally, the “Leave One Out Cross Validation” (LOOCV) validation method was implemented in all experiments because the method is deterministic. It is pertinent to clarify that the proposed method can currently only be implemented in binary classification datasets, but work is underway on a version to overcome this limitation.

#### 3.1.1 Algorithm

**Database and validation method**.

We assume a database with L numerical attributes and two classes:

- Class 1: D1 with n1 patterns.
- Class 2: D2 with n2 patterns.

The validation method involves splitting the database into two disjoint sets: a training set E and a test set P.

We will use Leave-One-Out Cross-Validation (LOOCV) due to its deterministic nature. To illustrate the proposed model, consider the Brain Cancer Kaggle database, where each pattern contains 7464 numerical attributes (L = 7,464):

- Class 1: D1 “Tumor” contains 18 patterns; that is, n1 = 18.
- Class 2: D2 “Normal” contains 18 patterns; that is, n2 = 18.

**Table:** 

70	81	25	10	22	113	36	…	138	53	-4	123	219

#### 3.1.2 First iteration of leave-one-out (example)

The first pattern of Class 1 (test pattern X) is included in the test set P.

P contains a single pattern: X = D1(1,:).

The rest of the patterns in the database form the training set E.

From this point on, throughout the learning phase, the information from pattern X will be excluded.

The set E contains the following:

- Y1: 17 patterns from Class 1 with L numeric attributes.
- Y2: 18 patterns from Class 2 with L numeric attributes.

Set E feed into the Learning Phase (note that test pattern X does not participate in this phase). At the output of the Learning Phase, three pieces of information are obtained:

- Position of Class 1.
- Learning attributes.
- Spherical frontiers vector.

**Figure 1:**
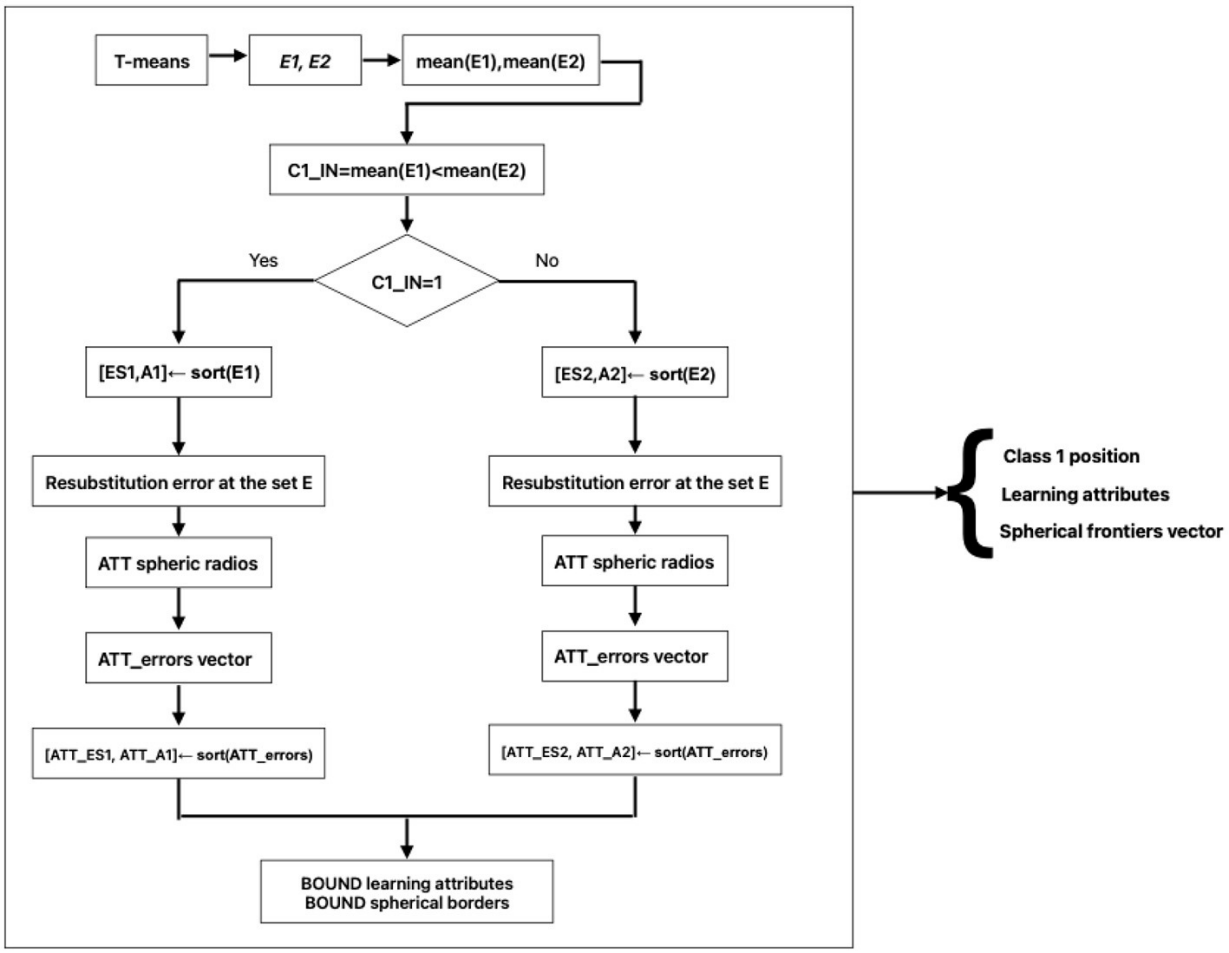
Training phase flow chart.

The first three steps are as follows: T-means, calculation of error vectors E1 and E2, and calculation of their arithmetic means. With mean(E1) and mean(E2), the value of C1_IN is obtained, which is crucial for the rest of the algorithmic steps. In the output of the Learning Phase, three pieces of very important information are obtained, which will be part of the input to the Classification Phase Figure 1.

In the first iteration of the Brain Cancer Kaggle example, the values of the parameters adjusted by Differential Evolution are:


ATT = 14 and BOUND = 5.

**Figure 2:**
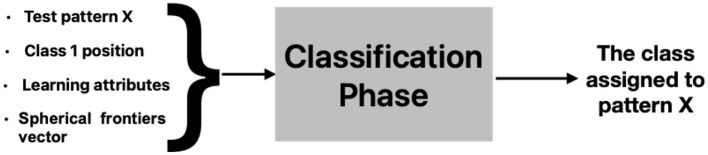
Classification phase.

For these three pieces of information, test pattern X is joined as input to the Classification Phase Figure 2.

At the output of the Classification Phase, the assigned class for the test pattern X is obtained. If X falls within the boundary defined by Class 1, it is assigned to Class 1; otherwise, it is assigned to Class 2.

#### 3.1.3 Training phase. Step 1: T-Means

One of the main challenges in minimalist machine learning is attribute selection. Although advancements like D-means (Molina, 2020) have been made, we propose a method with a different approach called Twice means or T-means, while also incorporating some elements from D-means. T-means consists of L iterations, one for each attribute j, from 1 to L.

- Y1: Projections of the training patterns from Class 1 onto attribute j (blue dots).
- Y2: Projections of the training patterns from Class 2 onto attribute j (red stars).

For each attribute j: T-means is the mean of the two means m1 and m2; m1 = mean(Y1); m2 = mean(Y2); T-means = mean ([m1 m2]).

**Figure 3:**
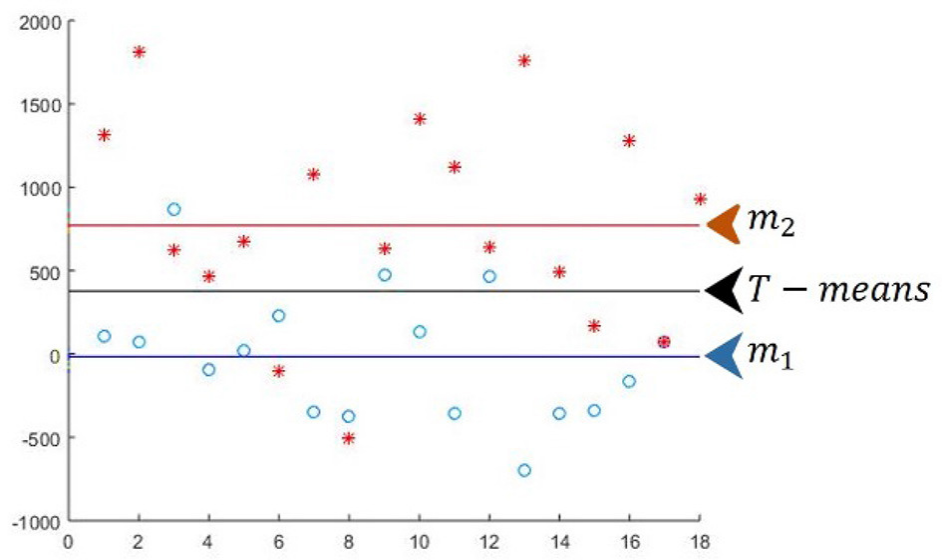
Diagram of the first iteration of T-means.

Diagram of the first iteration of T-means (j = 1), for the first iteration of the model (i = 1) Figure 3.

T_M is a vector with the L values of T-means. In this iteration of T-means, only attribute j = 1 is considered: T_M(1) = 376.3301.

#### 3.1.4 Learning Phase. Step 2: E1 y E2

E1 is a vector of L error values assuming that Class 1 is below T-means. For attribute j = 1, in E1(1) (Equation 8) there are 3 errors from Class 1 and 4 errors from Class 2:


(8) $$\begin{array}{c} E1\left(1\right)=3+4=7 \end{array}$$

E2 is a vector of L error values assuming that Class 1 is above T-means. For attribute j = 1, in E2(1) (Equation 9), there are 14 errors from Class 1 and 14 errors from Class 2:


(9) $$\begin{array}{c} E2\left(1\right)=14+14=28 \end{array}$$

**Figure 4:**
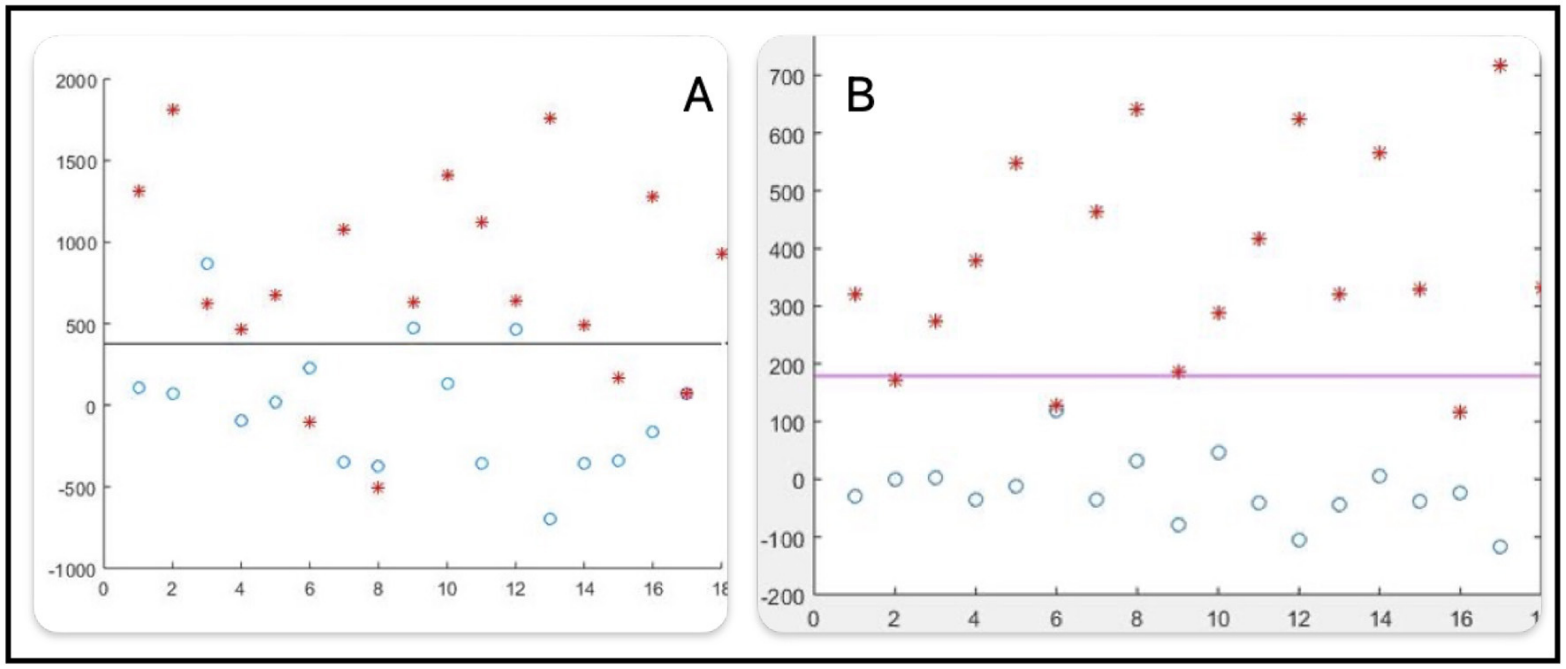
Diagram illustrating E1 and E2 for the first **(A)** and second **(B)** iterations of T-means.

Diagram illustrating Figure 4A. E1 and E2 for the first iteration of T-means, j = 1.

Diagram illustrating Figure 4B. E1 and E2 for the second iteration of T-means, j = 2.

For attribute j = 2, in E1(2) there are 0 errors from Class 1 and 3 errors from Class 2: E1(2) = 0 + 3 = 3.

For attribute j = 2, in E2(2) there are 17 errors from Class 1 and 15 errors from Class 2: E2(2) = 17 + 15 = 32.

#### 3.1.5 Learning Phase. Step 3: means of E1 and E2

E1 corresponds to the case where Class 1 is below T-means. In the example, E1 has L = 7,464 components.

**Table:** 

E1												
7	3	17	4	17	4	...	31	25	19	30	19	21

E2 corresponds to the case where Class 1 is above T-means. In the example, E2 has L = 7,464 components.

**Table:** 

E2												
28	32	18	31	18	31	...	4	10	16	5	16	14

The values of mean(E1) = 17.5355 and mean(E2) = 17.4606 clearly indicate the global behavior of E1 and E2.

#### 3.1.6 Learning Phase. Step 4.1: C1_IN criterion

**Figure 5:**
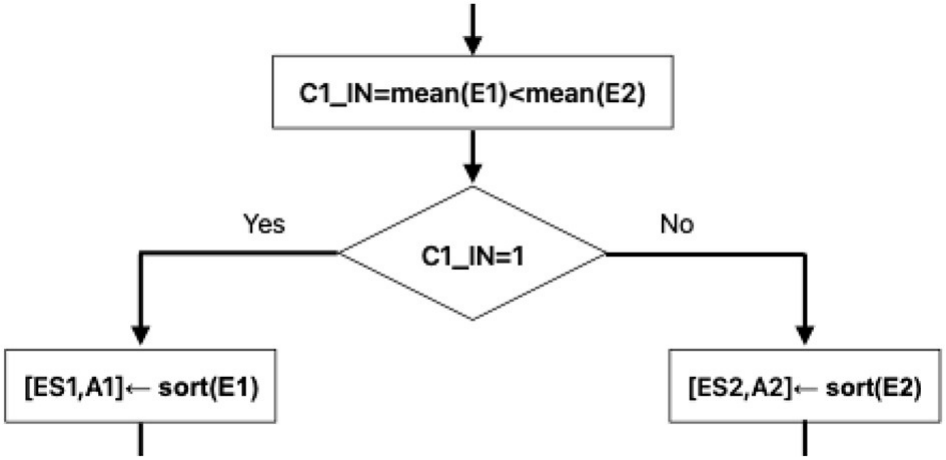
Decision criterion.

Early decision criterion for when the Classification Phase is reached ilustrated at the Figure 5.

#### 3.1.7 Step 4.2: two values of the criterion C1_IN

- The two possible values of C1_IN mark a branching point in the model.
- Two different paths are formed in the algorithmic steps.
- The value C1_IN = 1 indicates that in that path, the values of the vector E1 will be used.
- The value C1_IN = 1 indicates that in that path, the values of the vector E1 will be used.
- The value C1_IN = 0 indicates that in that path, the values of the vector E2 will be used.

The value of C1_IN indicates the correct position for the model to assign Class 1 to the test pattern X.


(10) $$\begin{array}{c} C1\text{\_}IN=mean\left(E1\right)<mean\left(E2\right) \end{array}$$

**Figure 6:**
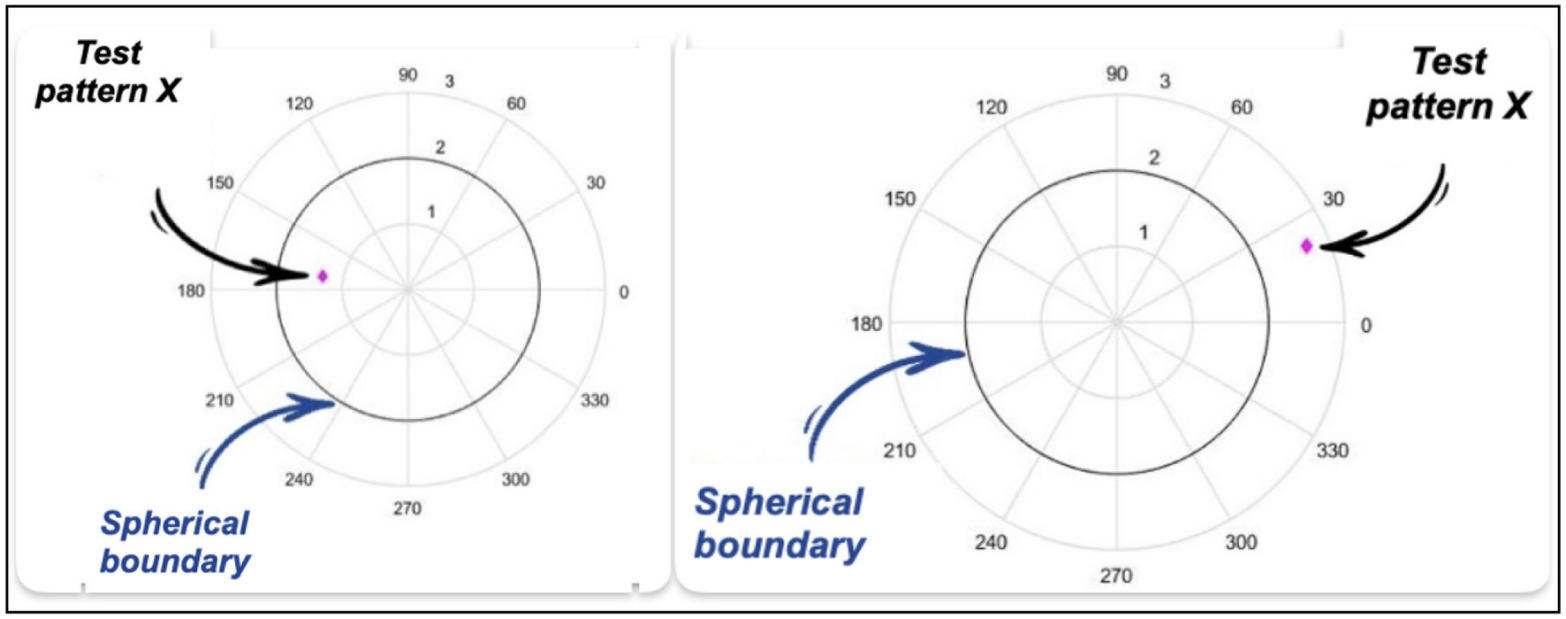
Point inside the spherical boundary and Point outside the spherical boundary.

If C1_IN = 1, Class 1 is assigned to the test pattern X if it is located inside the spherical boundary see Figure 6.

If X is located outside the spherical boundary, Class 2 is assigned.

If C1_IN = 0, Class 1 is assigned to the test pattern X if it is located outside the spherical boundary see Figure 6.

If X is located INSIDE the spherical boundary, Class 2 will be assigned.

#### 3.1.8 Learning Phase. Step 5: C1_IN = 0

Since C1_IN = 0, the values of the vector E2 will be used. In the example, E2 has L = 7,464 components.

**Table:** 

E2												
28	32	18	31	18	31	...	4	10	16	5	16	14

The vector of errors E2 is sorted:

[Sorted L values, Attribute indices]: [ES2, A2] ← sort(E2).

The first ATT attribute indices from A2 are selected (in the example, ATT = 14):

A2(1:14) = [7,176, 7,360, 7,383, 7,420, 7,428, 7,451, 2,573, 7,164, 7,220, 7,244, 7,299, 7,355, 7,389, 7,392].

**Resubstitution error in the training set**.

Output: ATT spherical radius.

**Figure 7:**
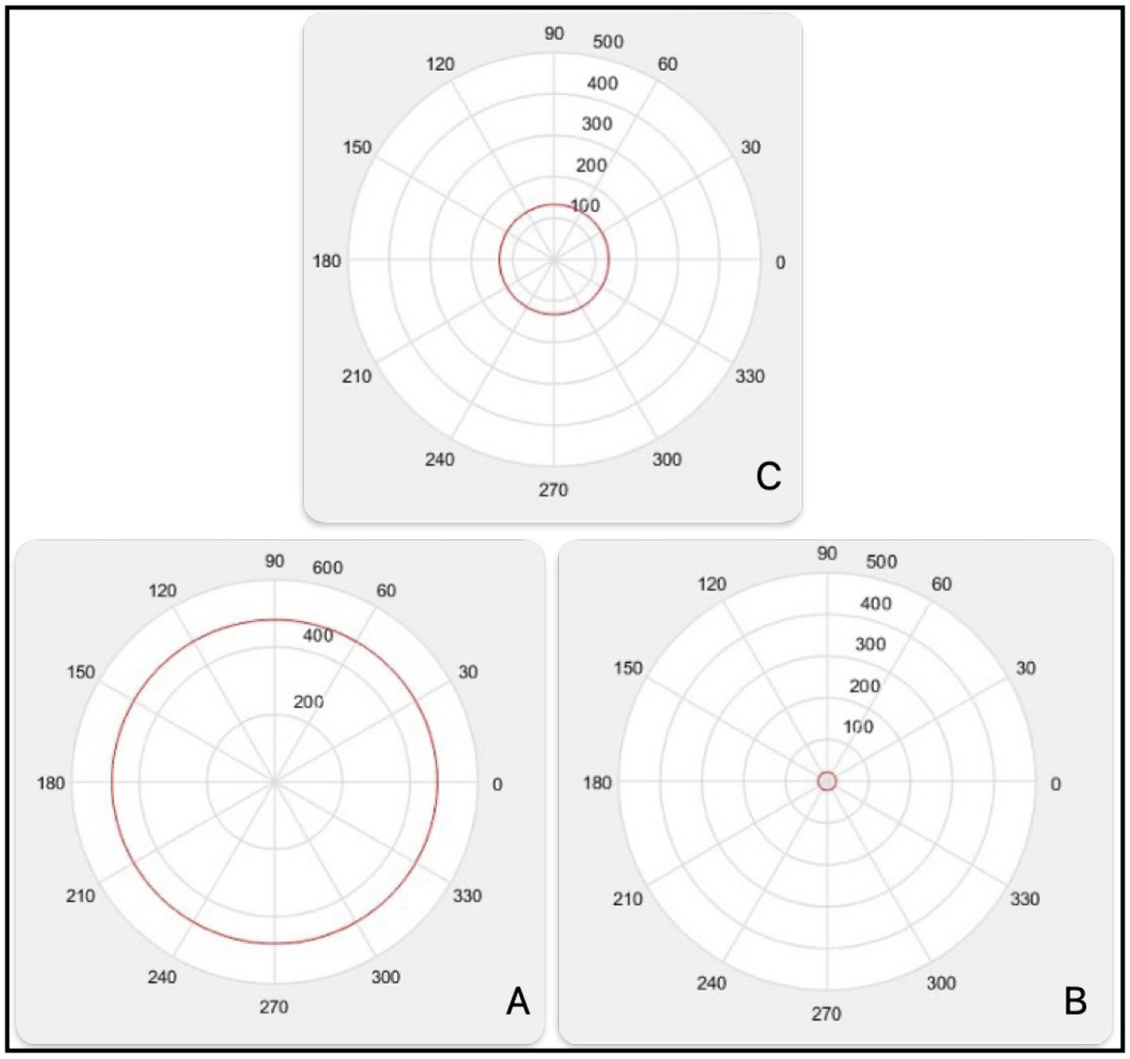
First, second and third spherical radius.

The first three spherical radius are illustrated in the Figure 7:


(11) $$\begin{array}{c} R\text{\_}esfA\left(7176\right)=480.5 \end{array}$$

(12) $$\begin{array}{c} R\text{\_}esfB\left(7360\right)=21.5 \end{array}$$

(13) $$\begin{array}{c} R\text{\_}esfC\left(7383\right)=132 \end{array}$$

The complete vector with the 14 spherical radius is:

R_esf= [480.5, 21.5, 132.5, 24, 25.5, 49, 33, 567.5, 356.5, 9.5, 459.5, 176, 226.5, 102.5].

##### 3.1.8.1 Resubstitution error in the training set

ATT spherical radius → ATT_errors vector.

**Figure 8:**
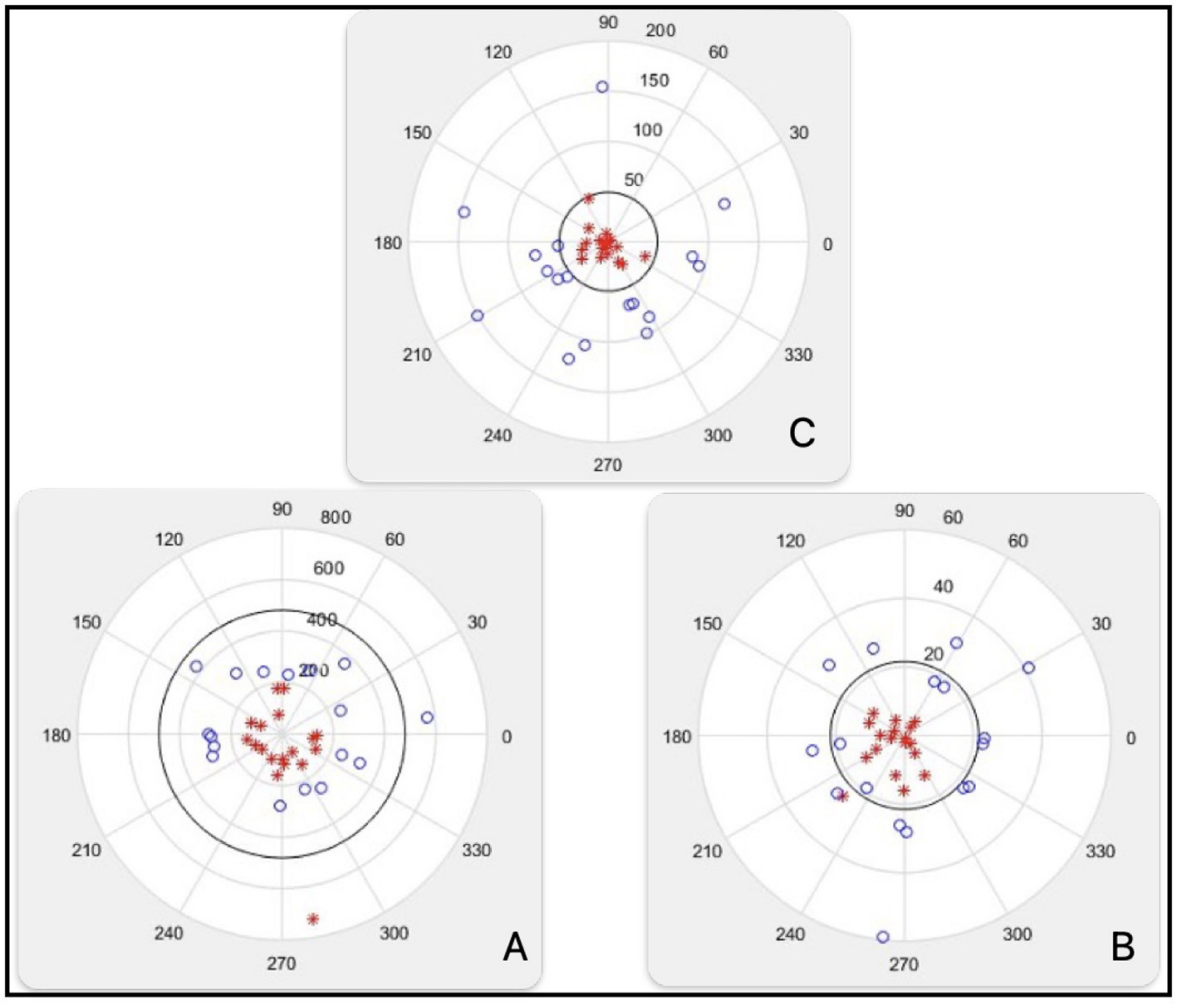
Patterns projected on A2 **(A)**, patterns projected on A2 **(B)**, class separated **(C)**.

In the Figure 8A, patterns projected onto attribute A2(1) = 7,176 are illustrated Figure 8A.

- The spherical radius (in black) is R_esf(7176) = 480.5.
- Since C1_IN=0C1_IN=0, Class 1 is OUTSIDE the spherical radius.
- Class 1 generates 16 errors (blue balls inside).
- Class 2 generates only 1 error (red star outside).
- ATT_errors(1)=16 + 1=17 errors.

At the same Figure 8B, patterns projected onto attribute A2(2) = 7,360 are illustrated.

- The spherical radius (in black) is R_esf(7,360) = 21.5.
- Class 1 generates 4 errors (blue balls inside).
- Class 2 generates only 1 error (red star outside).
- ATT_errors(2) = 4 + 1 = 5 errors.

In the same Figure 8C, patterns projected onto attribute A2(6) = 7,451 are illustrated.

- The spherical radius (in black) is R_esf(7,451) = 49.
- Class 1 and Class 2 are perfectly separated by the spherical radius Figure 8C.
- ATT_errors(6) = 0 errors.

Below is the complete Table 2 of attributes, spherical radius, and errors. The first iteration of the example, ATT = 14.

**Table 2:** First iteration.

**Attribute index**	**Spherical radius**	**Errors**
7,176	480.5	17
7,360	21.5	5
7,383	132.5	8
7,420	24.0	7
7,428	25.5	4
7,451	49.0	0
2,573	33.0	29
7,164	567.5	16
7,220	356.5	2
7,244	9.5	5
7,299	459.5	3
7,355	176.0	3
7,389	226.5	3
7,392	102.5	4

The ATT_errors vector is sorted:

- In [ATT_errors sorted, ATT_features_ord]: [ATT_ES1, ATT_A1] ← sort (ATT_errors).
- In [ATT_errors sorted] = [0, 2, 3, 3,3, 4, 4, 5, 5, 7, 8, 16, 17, 29].
- In [ATT_features_ord]=[7,451, 7,220, 7,299, 7,355, 7,389, 7,428, 7,392, 7,360, 7,244, 7,420, 7,383, 7,164, 7,176, 2,573].

For the first iteration of the example, BOUND = 5. Progressive sets are formed based on ATT_features_ord:

- SET_1 = 7,451.
- SET_2 = 7,451, 7,220.
- SET_3 = 7,451, 7,220, 7,299.
- SET_4 = 7,451, 7,220, 7,299, 7,355.
- SET_5 = 7,451, 7,220, 7,299, 7,355, 7,389.

Decision spherical BOUND is calculated:

For the first iteration of the example, BOUND = 5.

SET_1 = 7,451.

**Figure 9:**
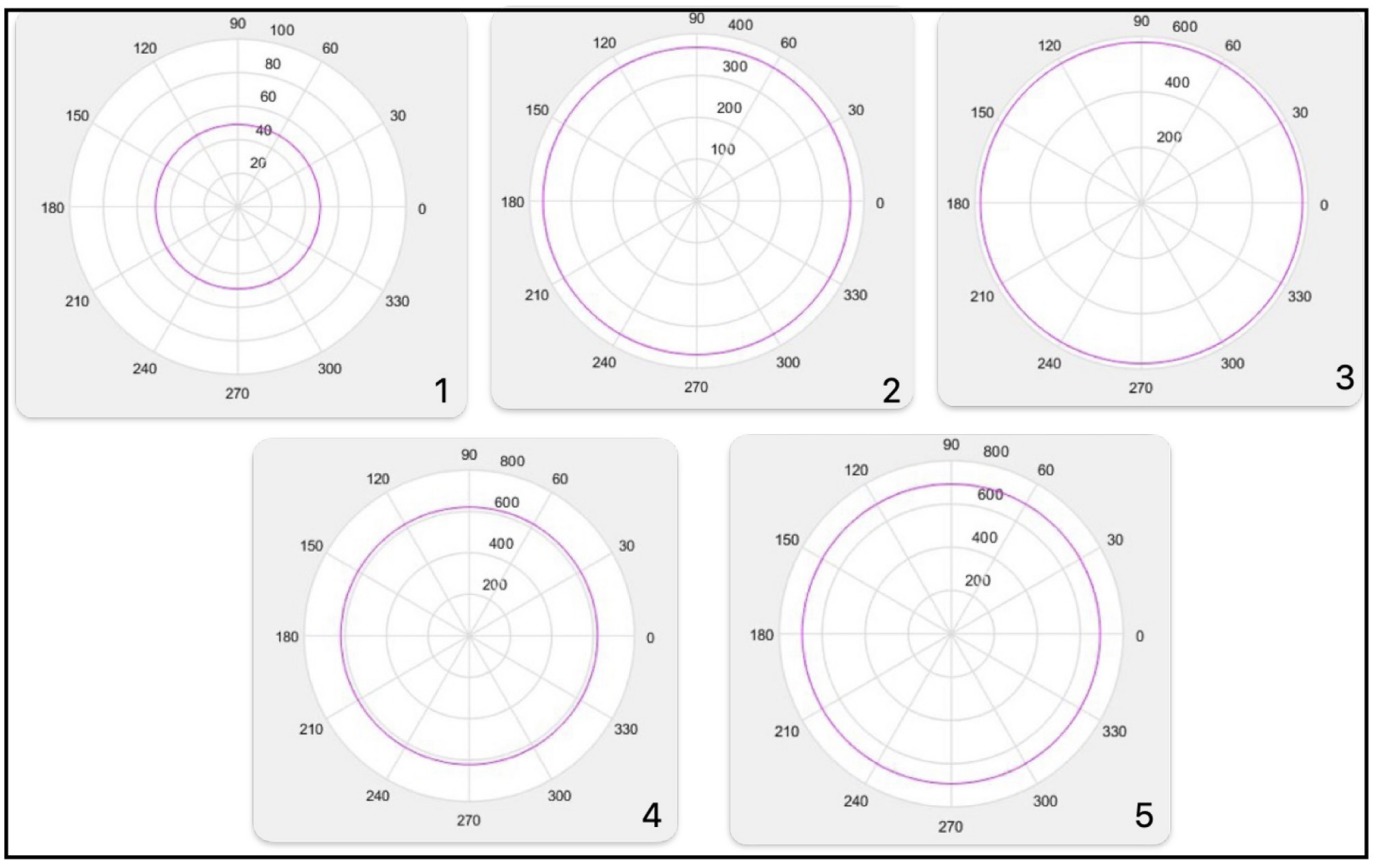
Decision spherical boundary 1, boundary 2, boundary 3, boundary 4, boundary 5.

Decision spherical boundary_1 = 49 Figure 9 No.1.

SET_2 = 7,451, 7,220.

Decision spherical boundary_2 = 367.4421 Figure 9 No.2.

SET_3 = 7,451, 7,220, 7,299.

Decision spherical boundary_3 = 579.4759 Figure 9 No.3.

For SET_3, the decision boundary is in 3D.

The image of this decision boundary is a sphere immersed in 3D. The figure illustrates a projection of this decision boundary in 2D.

SET_4 = 7,451, 7,220, 7,299, 7,355.

For SET_4, the decision boundary is in 4D. The image of this decision boundary is a sphere immersed in 4D.

The figure illustrates a projection of this decision boundary in 2D. Decision spherical boundary_4 = 621.0160 Figure 9 No.4.

SET_5 = 7,451, 7,220, 7,299, 7,355, 7,389.

For SET_5, the decision boundary is in 5D. The image of this decision boundary is a sphere immersed in 5D.

The figure illustrates a projection of this decision boundary in 2D. Decision spherical boundary_5 = 691.8988 Figure 9 No.5.

#### 3.1.9 Output of the Learning Phase

For the example at hand, the output of the Learning Phase is as follows:

The position of Class 1 is inside the decision boundary (C1_IN = 0).

Learning attributes: 7,451, 7,220, 7,299, 7,355, 7,389 (Boundary = 5).

Vector of spherical boundaries: [49, 367.4421, 579.4759, 621.0160, 691.8988].

#### 3.1.10 Output of the Learning Phase

- Step 1: Generate progressive sets from the learning attributes.
- Step 2: Project the test pattern onto the progressive attribute sets.
- Step 3: Calculate the spherical norm in each of the projections from Step 2.
- Step 4: Compare the spherical norms from Step 3 with the corresponding spherical boundaries.
- Step 5: Based on the value of C1_IN, decide the class of the test pattern.
  ∘ 5.1: C1_IN = 1 (correct Class 1 position is inside the spherical boundary).
    ▪ 5.1.1 If spherical norm < spherical boundary → assign Class 1 to the test pattern.
    ▪ 5.1.2 If spherical norm > spherical boundary → assign Class 2 to the test pattern.
  ∘ 5.2: C1_IN = 0 (correct Class 1 position is outside the spherical boundary).
    ▪ 5.2.1 If spherical norm > spherical boundary → assign Class 1 to the test pattern.
    ▪ 5.2.2 If spherical norm < spherical boundary → assign Class 2 to the test pattern.
- Step 6: Assign the class to the test pattern by voting on the results from Step 5.

Example. -

For the example at hand, the input to the Classification Phase is: Test pattern X = D1(1,:).

Since C1_IN = 0, the correct position for Class 1 is: outside the decision boundary.

Learning attributes: 7,451, 7,220, 7,299, 7,355, 7,389.

Spherical boundary vector: [49, 367.4421, 579.4759, 621.0160, 691.8988].

Votes for Class 1: 5.

Votes for Class 2: 0.

Class 1 is assigned to the test pattern X = D1(1,:) see Table 3.

**Table 3:** Class assigned.

**Progressive sets**	**Projections at X**	**Spherical norms**	**Spherical boundaries**	**Vote**
{7,451}	X'=[108]	108.0000	49.0000	Class 1
{7,451, 7,220}	X'=[108 425]	438.5077	367.4421	Class 1
7,451, 7,220, 7,299	X'=[108 425 797]	909.6692	579.4759	Class 1
{7,451, 7,220, 7,299, 7,355}	X'=[108 425 797 255]	944.7344	621.0160	Class 1
{7,451, 7,220, 7,299, 7,355, 7,389}	X'=[108 425 797 255 83]	962.2952	691.8988	Class 1

### 3.2 N-Spherical MML model pseudocode and computational cost

The pseudocode for the N-Spherical MML model consists of three main phases:

- Learning Phase.
- Parameter Self-Adjustment using Metaheuristics.
- Classification Phase.

#### 3.2.1 Learning Phase

**Signature:** Learn (*Y, att, Boundary*)

**Inputs:**

- *Y*: Training set with instances described by *n* + 1 attributes.
- *att*: Percentage of attributes to select.
- *Boundary*: Maximum limit for sets of attributes used in spherical radii.

**Outputs:**

- *C*1_*IN*: Boolean value indicating the spatial position of the classes.
- *Edic*_*esf*: Dictionary of ordered attributes.
- *R*1: Vector of spherical radii.
- *e*: Training error.

**Steps:**

1. Divide the training set *Y* into two subsets *Y*1 and *Y*2, where *Y*1 ← {*y* ∈ *Y*:*y*[*n*] = 1} and *Y*2 ← {*y* ∈ *Y*:*y*[*n*] = 2}. The value of the vector *y* at position *n* denotes the class of the pattern. ***O(m)***.
2. Let *t*1 and *t*2 represent the number of instances in *Y*1 and *Y*2, respectively.
3. Create five arrays of size *n* − 1: *m*1, *m*2, *Tm, E*1, and *E*2. ***O(1)***.
4. For *i* = 0…*n* − 1 (for each attribute) ***O(n)***.
  (a) *m*1[*i*] ← mean(*Y*1[*i*]). (Calculates the mean of the values in column *i* of matrix *Y*1). ***O(m)***.
  (b) *m*2[*i*] ← mean(*Y*2[*i*]). ***O(m)***.
  (c) *Tm*[*i*] ← (*m*1[*i*]+*m*2[*i*])/2. ***O(1)***.
  (d) *E*1[*i*] ← sum(*Y*1[*i*] > *Tm*[*i*])+sum(*Y*2[*i*] < *Tm*[*i*]). ***O(m)***. (The total number of errors committed is counted, considering that class 1 is BELOW class 2) ***O(m)***.
  (e) *E*2[*i*] ← sum(*Y*1[*i*] < *Tm*[*i*])+sum(*Y*2[*i*] > *Tm*[*i*]). (The total number of errors committed is counted, considering that class 1 is ABOVE class 2) ***O(m)***.
5. *C*1_*IN* ← (mean(*E*1) < mean(*E*2)) (decides if class 1 is BELOW (*C*1_*IN* = 1) or ABOVE (*C*1_*IN* = 0) in spherical coordinates). ***O(1)***.
6. If *C*1_*IN* = 1, create a sorted dictionary *Edic* (key = position, value = error count) using *E*1, ordered by ascending error counts. ***O(1)***.
7. Else, create *Edic* using *E*2, ordered by ascending error counts. ***O(1)***.
8. Calculate *c* ← (att × *n*)/100. ***O(1)***.
9. Remove the last *n-c* records from *Edic*, retaining only the selected attributes. ***O(n)***.
10. Generate new matrices *Y*1′ and *Y*2′ using attributes in *Edic*:
  - For *i* = 0…*t*1 − 1, *j* = 0…*c* − 1: ***O*****(*n* · *m*)**.
    ∘ *Y*1′[*i*][*j*] ← *Y*1[*i*][Edic.Key[j]].
    ∘ *Y*1′[*i*][*c*] ← 1.
  - For *i* = 0…*t*2 − 1, *j* = 0…*c* − 1: ***O*****(*n* · *m*)**.
    ∘ *Y*2′[*i*][*j*] ← *Y*2[*i*][Edic.Key[j]].
    ∘ *Y*2′[*i*][*c*] ← 2.
11. Convert *Y*1′ and *Y*2′ to spherical coordinates (*Y*1′*e, Y*2′*e*). ***O*****(*n* · *m*)**.
12. Create two vectors *r*1, *r*2 of size *t*1 and *t*2, respectively. ***O(1)***.
13. For *i* = 0…*c* − 1 (for each attribute to calculate spherical radii): ***O(n)***.
  - For *j* = 0…*t*1 − 1: ***O(m)***
    ∘ $r1\left[j\right]\leftarrow \sqrt{\overset{i}{\underset{k=0}{\sum }}{\left(Y1^{\prime }e\left[j\right]\left[k\right]\right)}^{2}}$.
  - For *j* = 0…*t*2 − 1:
    ∘ $r2\left[j\right]\leftarrow \sqrt{\overset{i}{\underset{k=0}{\sum }}{\left(Y2^{\prime }e\left[j\right]\left[k\right]\right)}^{2}}$.
14. If *C*1_*IN* = 1:
  - Compute *R*_*frontier*[*i*] ← (max(*r*1[*i*])+min(*r*2[*i*]))/2. ***O(n)***.
  - Calculate substitution errors:
$$\begin{array}{r} \text{errors }[i]\leftarrow \text{sum }(r1\;[i]>R\_frontier\;[i])+\text{sum }(r2\;[i]\\ <R\_frontier\;[i]).O(n\cdot m). \end{array}$$
15. Else:
  - Compute *R*_*frontier*[*i*] ← (min(*r*1[*i*]) + max(*r*2[*i*]))/2.
  - Calculate substitution errors:
$$\begin{array}{r} \text{errors }[i]\leftarrow \text{sum }(r1\;[i]<R\_frontier\;[i])+\text{sum }(r2\;[i]\\ >R\_frontier\;[i]). \end{array}$$
16. Create a sorted dictionary *Edic*_*esf* (key = position, value = error count) using errors, ordered by ascending error counts. ***O(1)***.
17. Assign *e* ← errors[0] (minimum error). ***O(1)***.
18. For *i* = 0…Boundary − 1: ***O(n)***.
  - Create attribute subset SET with the first *i* attributes.
  - Update *r*1[*j*], *r*2[*j*] for each instance in *Y*1′*e, Y*2′*e*. ***O*****(*n* · *m*)**.
19. Return *C*1_*IN, Edic*_*esf, R*1, *e*.

**Total cost:** The complexity is cubic, assuming *n* ≫ *m*.

**Approximated as**


$$\begin{array}{c} O\left(2n^{2}m+4mn+m+n\right)\approx O\left(2n^{3}+4n^{2}+2n\right). \end{array}$$

**Note:** Terms with ***O(1)*** are not considered in the calculation, since the focus is on the upper bound, representing the worst-case cost.

#### 3.2.2 Parameter self-adjustment using metaheuristics

**Signature:** AutoLearn(*Y, np, F, pR*).

**Inputs:**

- *Y*: Training set with instances described by *n* + 1 attributes.
- *np*: Population size.
- *F*: Mutation factor.
- *pR*: Recombination probability.
- *G*: Number of generations.

**Outputs:**

- *C*1_*IN, Edic*_*esf, R*1, *e* : Optimal parameters and training results.
- [*att, Boundary*]: Adjusted parameter values.

**Steps:**

1. Create an empty dictionary *P* to store the population and fitness of individuals (*Key = individual, Value = optimization value*). ***O(1)***.
2. For *i* = 0…*np* − 1 (for each individual): ***O(np)***.
  (a) Create a two-dimensional integer vector *x*_*i*_ with values bounded by *n*. ***O(1)***.
  (b) Add the vector *x*_*i*_ to *P*.*Key*. ***O(1)***.
  (c) Evaluate the vector *x*_*i*_ by assigning it an optimization value *f*_*i*_ = Learn(*Y, x*_*i*_[1], *x*_*i*_[2]). ***O*****(2*n*^3^ + 4*n*^2^ + 2*n*)**.
  (d) Add the optimization value to *P*.*Value*. ***O(1)***.
3. For gen = 1…*G* (for each generation): ***O(G)***.
  - For *i* = 0…*np* − 1 (for each individual): ***O(np)***.
    (a) Randomly select three vectors, denoted as *x*_*a*_, *x*_*b*_, *x*_*c*_, such that *a* ≠ *b* ≠ *c* ≠ *i*. ***O(1)***.
    (b) Create a new individual *n*_*i*_ = *x*_*c*_ + *F*(*x*_*a*_ − *x*_*b*_). ***O(1)***.
    (c) If rand(0, 1) < pR:
      i. Calculate $f_{i}^{\prime }=\text{Learn}\left(Y,n_{i}\left[1\right],n_{i}\left[2\right]\right)$. ***O*****(2*n*^3^ + 4*n*^2^ + 2*n*)**.
      ii. If $f_{i}^{\prime }.e>f_{i}.e$, replace *x*_*i*_ with *n*_*i*_ in the list *P*. ***O(1)***.
4. Sort the dictionary *P* in ascending order of optimization values (since these are errors, smaller values come first). ***O*****(*np*^2^)**.
5. Return the optimization value of the first individual in the population (*C*1_*IN, Edic*_*esf, R*1, *e*) and the first individual's vector (parameter values [*att, Boundary*]).

**Total cost:** The complexity remains cubic, as the previous phase was also cubic.

Approximated as


$$\begin{array}{r} O\left(np\left(2n^{3}+4n^{2}+2n\right)\right)+O\left(G\cdot np\left(2n^{3}+4n^{2}+2n\right)\right)+O\left(np^{2}\right)\\ \approx O\left(np\left(2n^{3}+4n^{2}+2n\right)\left(1+G\right)+np^{2}\right). \end{array}$$

**Assumptions:** Since *np* ≪ *n* and *G* ≫ 1, the complexity is bounded as follows:


$$\begin{array}{c} O\left(n\left(2n^{3}+4n^{2}+2n\right)\cdot G+n^{2}\right)\approx O\left(2Gn^{4}+4Gn^{3}+n^{2}\left(G+1\right)\right). \end{array}$$

**Note:** Terms with ***O*****(1)** are disregarded in the calculation as we focus on the upper bound, representing the worst-case cost.

#### 3.2.3 Classification phase

**Signature:** *Classif*(*p*)

**Inputs:**

- *p*: Instance to classify, described by *n* + 1 attributes.

**Outputs:**

- *class*: Assigned class label for the instance.

**Steps:**

1. Obtain a new instance *p*′, considering the attributes present in *Edic*_*esf*, in the order they appear in the dictionary, as follows:
  - For *i* = 0…*c* − 1 (for each attribute to consider): ***O*****(*n*)**.
    ∘ *p*′[*i*] ← *p*[*Edic*_*esf*.*Key*[*i*]].
2. Create a boolean vector *votes* of size Boundary, initialized to False. ***O*****(1)**.
3. For *i* = 0…Boundary − 1 (for each attribute to consider): ***O*****(*n*)**.
  (a) Create the attribute set SET with the first *i* attributes.
  (b) Initialize temp ← 0.
  (c) For *j* = 0…*i* (to compute the spherical radius): ***O*****(*n*)**.
    - temp ← temp + (*p*′[*j*])^2^.
  (d) $r1\left[i\right]\leftarrow \sqrt{\text{temp}}$.
  (e) If *R*1[*i*] < *r*1[*i*]∧*C*1_*IN*, then: ***O*****(1)**.
    - *votes*[*i*] ← True.
4. Count the votes for class 1 as votes_c1 = |{*votes*[*i*] = True:*i* = 1…Boundary}|. ***O*****(*n*)**.
5. If votes_c1 ≥ Boundary/2, then:
  - class ← 1.
6. Otherwise:
  - class ← 2.
7. Return class.

**Total cost:** Quadratic in the worst case where att = Boundary = *n*.

Approximated as


$$\begin{array}{c} O\left(n\right)+O\left(n^{2}\right)+O\left(n\right)\approx O\left(n^{2}\right)+O\left(2n\right). \end{array}$$

### 3.3 Balance accuracy

To verify the usability of the proposed model on imbalanced datasets, balanced accuracy results were reviewed, as shown in Table 4.

**Table 4:** Balanced accuracy.

	**Naive bayes**	**MLP**	**J48**	**Logistic**	**SMO**	**IB1**	**N-spherical**
AP endometrium kidney	0.9860	0.5000	0.9334	0.9884	0.9860	0.9860	0.9961
AP lung kidney	0.9748	0.5042	0.9194	0.9707	0.9765	0.9626	0.9763
AP endometrium prostate	0.9691	0.4373	0.9845	0.9845	0.9855	0.9918	0.9927
AP breast uterus	0.9462	0.5000	0.9327	0.9528	0.9410	0.9356	0.9567
AP breast ovary	0.9615	0.4907	0.9254	0.9627	0.9671	0.9245	0.9414
AP ovary lung	0.9101	0.5129	0.9216	0.9249	0.9155	0.9057	0.9260
Brain cancer kaggle	0.8888	0.9722	0.8888	0.9722	0.9722	0.9444	1.0000
Lung	0.6612	0.4966	0.3911	0.6350	0.5604	0.5147	0.8534
Lymphoma	0.9327	0.9555	0.8248	0.9337	0.9327	0.7288	0.9772
Leukemia	0.8636	1.0000	0.9359	0.9359	1.0000	1.0000	1.0000
Nutt	0.7500	0.7857	0.8214	0.8928	0.9285	0.6071	0.9642
Diabetic mellitus	0.9031	0.8806	0.9945	0.9238	0.8958	0.5981	0.9366
Covid-19 kaggle	0.9309	0.9745	0.9812	0.9454	0.9189	0.9727	0.9911

With the Balanced Accuracy performance metric:

- The proposed N-Spherical MML model ranks first in ten datasets.
- SMO ranks first in three datasets.
- IB1, MLP, and J48 each rank first in only one dataset.

To investigate whether there are significant differences in performances, the Friedman test was employed. The ranking obtained is as follows, demonstrating that the proposed Spherical MML model ranks first with a value of 1.5 compared to the remaining six algorithms. This establishes it as the best model for the classification task described in this article document.

Conversely, the Naïve Bayes algorithm ranks last in the Friedman test ranking table, with a value of 4.6154 see Table 5.

**Table 5:** Balanced accuracy friedman ranking.

**Algorithm**	**Ranking**
Naive bayes	4.6154
MLP	5.3462
J48	4.8077
Logistic	3.2308
SMO	3.4315
IB1	5.0385
N-spherical	1.5

The Friedman test results indicate that the null hypothesis is rejected with a confidence level of 95% and a *p-*value of 0.0000287. Therefore, significant differences exist between the classifiers. The Holm post-test was applied to identify which classifiers exhibit statistically significant differences. The test rejects the hypothesis with an adjusted *p*-value of ≤ 0.05 see Table 6.

**Table 6:** Balanced accuracy holm post-test.

**I**	**Algorithm**	***z* = (*R*_0_ − *R*_*i*_)/*SE***	**p**	**Holm**
6	MLP	4.539206	0.000006	0.008333
5	IB1	4.17607	0.00003	0.01
4	J48	3.903718	0.00095	0.0125
3	Naive Bayes	3.676757	0.000236	0.016667
2	SMO	2.314995	0.020613	0.025
1	Logistic	2.042643	0.041088	0.05

The proposed model in this article, the Spherical MML, is significantly better than Logistic, SVM-SMO, Naïve Bayes, J48, IB1, and MLP.

## 4 Discussion

The N-Spherical MML classifier, developed within the Minimalist Machine Learning (MML) paradigm, introduces a novel approach to tackling challenges in pattern classification. This model has demonstrated significant effectiveness and robustness when applied to complex datasets, particularly those characterized by high dimensionality and class imbalance. By leveraging its minimalist design, the N-Spherical MML classifier achieved substantial improvements in balanced accuracy, consistently outperforming state-of-the-art classifiers in various scenarios.

When evaluated across 13 datasets, the N-Spherical MML ranked first in 10 cases, showcasing its ability to generalize and adapt across diverse datasets, particularly in the domain of disease-related data. Statistical analyses, including the Friedman and Holm tests, confirmed that the model provides statistically significant advantages over widely adopted classifiers such as Naïve Bayes, J48, Logistic Regression, SVM-SMO, IB1, and MLP. These findings underscore the potential of N-Spherical MML as a robust and reliable tool for classification tasks, particularly in challenging scenarios involving imbalanced and high-dimensional datasets.

Despite its promising results, the proposed model has several limitations that must be addressed in future research. Currently, it is restricted to binary classification tasks, which significantly limits its applicability in real-world scenarios where multi-class datasets are prevalent. Additionally, while the model effectively handles numerical data, it does not support categorical or mixed data types, which are common in many application domains. Addressing these limitations is critical for extending the model's applicability and ensuring its relevance across a broader range of datasets.

In terms of computational complexity, the model has been analyzed with respect to memory usage and execution time. While the N-Spherical MML is scalable for datasets of moderate size, its performance on larger datasets could benefit from further optimization. Exploring alternative metaheuristic algorithms and transformations represents another opportunity for enhancing the model's performance and efficiency.

### 4.1 Conclusions

The N-Spherical MML classifier represents a significant advancement within the Minimalist Machine Learning paradigm, providing a robust and effective solution for binary classification tasks involving high-dimensional and imbalanced datasets. By achieving first-place performance in 10 out of 13 datasets and demonstrating statistically significant advantages over widely used classifiers, the model has established its potential as a powerful tool in machine learning.

However, the model's limitations—its current restriction to binary classification and its lack of support for categorical or mixed data types—highlight important areas for future work. Additionally, further optimization of computational complexity and the exploration of alternative metaheuristic algorithms could enhance its scalability and efficiency for larger datasets.

In summary, the N-Spherical MML classifier delivers strong classification performance and demonstrates the versatility of the minimalist approach to machine learning. By addressing its current limitations, this model could evolve into a more comprehensive solution applicable to a broader range of real-world problems, further solidifying its contribution to the field of machine learning.

### 4.2 Future work

To further improve the N-Spherical MML classifier, several avenues of future research are proposed:

1. **Enhanced transformations:** Develop a transformation method that surpasses the effectiveness of T-means, potentially improving the model's adaptability to diverse datasets.
2. **New classification criteria:** Propose alternative criteria to replace C1_IN, which determines the spatial positioning of classes, to increase classification accuracy.
3. **Multi-class support:** Extend the model to handle datasets with more than two classes, increasing its applicability in real-world multi-class classification problems.
4. **Support for categorical data:** Enhance the model's capabilities to process both numerical and categorical data, enabling its use in mixed-type datasets.
5. **Exploration of metaheuristics:** Investigate and incorporate metaheuristic algorithms beyond Differential Evolution to further optimize the parameter selection process.

These improvements will address the current limitations of the N-Spherical MML model, enabling it to tackle a broader range of classification problems and datasets with higher complexity. By following these directions, the model can become a more versatile and powerful tool for machine learning practitioners.

## Data Availability

The original contributions presented in the study are included in the article/supplementary material, further inquiries can be directed to the corresponding author.

## Footnotes

^1^OpenML (Unknown). *lymphoma_2classes*. Dataset. Available at: https://www.openml.org/d/1101 (accessed November 20, 2024).

^2^JillaniSoftTech (Unknown). *Brain tumor*. Dataset. Available at: https://www.kaggle.com/datasets/jillanisofttech/brain-tumor (accessed November 20, 2024).
